# Enhancing dendrobine production in *Dendrobium nobile* through mono-culturing of endophytic fungi, *Trichoderma longibrachiatum* (MD33) in a temporary immersion bioreactor system

**DOI:** 10.3389/fpls.2024.1302817

**Published:** 2024-01-29

**Authors:** Surendra Sarsaiya, Archana Jain, Fuxing Shu, Mingfa Yang, Mengxuan Pu, Qi Jia, Qihai Gong, Qin Wu, Xu Qian, Jingshan Shi, Jishuang Chen

**Affiliations:** ^1^ Key Laboratory of Basic Pharmacology and Joint International Research Laboratory of Ethnomedicine of Ministry of Education, Zunyi Medical University, Zunyi, China; ^2^ Bioresource Institute for Healthy Utilization (BIHU), Zunyi Medical University, Zunyi, China; ^3^ College of Biotechnology and Pharmaceutical Engineering, Nanjing Tech University, Nanjing, China

**Keywords:** dendrobine, *Dendrobium nobile*, endophytic fungi, temporary immersion bioreactor system (TIBS), alkaloid production

## Abstract

**Introduction:**

Dendrobine, a valuable alkaloid found in *Dendrobium nobile*, possesses significant pharmaceutical potential.

**Methods:**

In this study, we explored innovative approaches to enhance dendrobine production by utilizing endophytic fungi in a Temporary Immersion Bioreactor System (TIBS, Nanjing BioFunction Co. Ltd., China) and traditional test bottles. Dendrobine was unequivocally identified and characterised in *D. nobile* co-culture seedlings through UHPLC analysis and LC-MS qTOF analysis, supported by reference standards.

**Results:**

The CGTB (control group) and EGTB (experimental group) 12-month-old *D. nobile* seedlings exhibited similar peak retention times at 7.6±0.1 minutes, with dendrobine identified as C_16_H_25_NO_2_ (molecular weight 264.195). The EGTB, co-cultured with *Trichoderma longibrachiatum* (MD33), displayed a 2.6-fold dendrobine increase (1804.23 ng/ml) compared to the CGTB (685.95 ng/ml). Furthermore, a bioanalytical approach was applied to investigate the mono-culture of *T. longibrachiatum* MD33 with or without *D. nobile* seedlings in test bottles. The newly developed UHPLC-MS method allowed for dendrobine identification at a retention time of 7.6±0.1 minutes for control and 7.6±0.1 minutes for co-culture. Additionally, we explored TIBS to enhance dendrobine production. Co-culturing *D. nobile* seedlings with *Trichoderma longibrachiatum* (MD33) in the TIBS system led to a substantial 9.7-fold dendrobine increase (4415.77 ng/ml) compared to the control (454.01 ng/ml) after just 7 days. The comparative analysis of dendrobine concentration between EGTB and EGTIBS highlighted the remarkable potential of TIBS for optimizing dendrobine production. Future research may focus on scaling up the TIBS approach for commercial dendrobine production and investigating the underlying mechanisms for enhanced dendrobine biosynthesis in *D. nobile*. The structural elucidation of dendrobine was achieved through ^1^H and ^13^C NMR spectroscopy, revealing a complex array of proton environments and distinct carbon environments, providing essential insights for the comprehensive characterization of the compound.

**Discussion:**

These findings hold promise for pharmaceutical and industrial applications of dendrobine and underline the role of endophytic fungi in enhancing secondary metabolite production in medicinal plants.

## Introduction

*Dendrobium nobile*, an enduring epiphytic herb belonging to the Orchidaceae family, holds a significant place in traditional Chinese medicine. This remarkable plant is primarily found in regions south of the Yangtze River, encompassing Guizhou, Yunnan, Guangxi, and other sub-tropical areas. With a global diversity of over 25,000 plant species within the *Dendrobium* genus (Nie et al., 2020; Sarsaiya et al., 2020a; Sarsaiya et al., 2020b), *Dendrobium nobile* stands out for its invaluable contributions to the history of drug development. Among its many compounds, dendrobine is a noteworthy alkaloid found in *Dendrobium nobile*, typically comprising about 0.5% of its total weight. This colorless solid, reminiscent of the picrotoxin family of natural products, has thus far been exclusively sourced from *D. nobile* and through chemical synthesis (Sarsaiya et al., 2020b). Over the decades, dendrobine has been harnessed for its therapeutic potential in addressing a range of health concerns. It has been explored in the treatment of conditions such as tumors, hyperglycemia, and hyperlipidemia and has displayed promising effects in mitigating metastatic cancer. Additionally, it has shown potential for addressing ailments associated with ageing, including Alzheimer’s disease and nervous system disorders (Nie et al., 2020; Sarsaiya et al., 2020b). However, the production of dendrobine by the *D. nobile* plant species is limited due to its slow growth rate. Consequently, the quantity of dendrobine derived from *D. nobile* remains insufficient to meet the current demands of both the industrial and research sectors. Robust and thriving plants host intricate and diverse microbial ecosystems that wield significant influence over the physiology and growth of these plants. In parallel, the variety within these plant-associated microbial populations is intricately intertwined with external environmental conditions. Beyond their primary role in nourishing plants, plant metabolites serve multifaceted functions, encompassing defence mechanisms against pathogens, pests, and herbivores (Zhao et al., 2023).

Most of the previous research pertaining to orchid-fungus associations has primarily focused on the fungal dynamics within terrestrial and temperate orchids. However, the intricate interplay between fungi and tropical orchids, exemplified by *D. nobile*, extends beyond the norm. Diverse fungal taxa inhabit various parts of *D. nobile*, encompassing roots, stems, buds, and leaves (Chen et al., 2023a). This rich fungal community includes mutualistic mycorrhizal partners as well as endophytic fungi, alongside a notably varied assembly of nonmycorrhizal fungal associates. Despite this breadth of association, the precise role these plant-associated fungi play in stimulating phytochemical production remains enigmatic (Sarsaiya et al., 2020a). In addition to their intriguing ecological roles, endophytic fungi confer substantial benefits upon their host, *D. nobile*. These advantages range from assisting the plant in coping with both biotic and abiotic stresses to strengthening its resistance against pathogens. This was accomplished through the secretion of secondary metabolites boasting antimicrobial properties and various phytohormones, as elucidated by Hajong and Kapoor (2020). Existing exploration of Dendrobium myco-endophytes (DMEs) has predominantly centered on *in vitro* symbiotic practices, utilizing fungal endophyte strains isolated from fully matured Dendrobium roots, buds, stems, and leaves. Unravelling the nature of these endophytic fungi—whether they assume pathogenic, conditionally pathogenic, or non-pathogenic roles for the host plant—carries significant importance (Sarsaiya et al., 2019). Nonetheless, the scope of orchid seed germination in its natural habitat likely entails a more intricate tapestry of interactions, potentially involving a broader array of non-mycorrhizal fungi and even other organism types, as posited by Salazar et al. (2020) and Hernández-Ramírez et al. (2023).

In the realm of plant-fungal co-culturing, a well-recognized phenomenon is the dormancy of numerous gene clusters. These silent gene clusters encode secondary metabolites that, under conventional laboratory culture conditions, remain unexpressed and, consequently, unproduced. To unlock the potential of these hidden metabolites, researchers have devised strategies to induce their expression. One such promising method involves co-culturing endophytic fungi with plants in the same growth medium. This intriguing approach capitalizes on the synergy between plant and fungal species, potentially leading to the activation of gene clusters and the subsequent production of metabolites (Chen et al., 2023b). In the natural world, microorganisms coexist within intricate communities, engaging in a myriad of interactions. Central to these interactions are the secondary metabolites that serve as chemical signals for communication or as tools in the competition for limited resources. These metabolites also play a pivotal role in fortifying defence mechanisms. Thus, emulating the complexity of the natural environment through the mixed fermentation of different microorganisms, often referred to as co-cultivation or co-culture, holds the promise of amplifying compound production. Moreover, this co-cultivation strategy may catalyze the awakening of dormant biosynthetic pathways, ultimately resulting in the accumulation of novel natural products (Akone et al., 2016; Gegenbauer et al., 2023).

To overcome these limitations, researchers have explored the possibility of cultivating microorganisms under conditions that mimic the complex communities found in nature, commonly referred to as the “microbiome.” Within these microbial communities, bioactive secondary metabolites assume a pivotal role in mediating interactions among various microorganisms and may have a direct influence on the production of phytochemicals in plants (Vinale et al., 2017; ZhiPing et al., 2023). Although previous studies have explored how *D. nobile* and endophytic fungi interact in terms of acquiring nutrients, promoting growth, and providing protection against pathogens, the specific contribution of these fungi in co-cultivation to boost the production of natural compounds has not been extensively investigated. In this study, we undertook the collection and analysis of *D. nobile* seedlings to quantify dendrobine concentrations. We conducted this analysis using LC-MS chromatography for two groups: the control group, consisting of *D. nobile* seedlings grown under standard conditions, and the experimental group, involving the co-culturing of *D. nobile* with *Trichoderma longibrachiatum* MD33. Importantly, this co-culturing was conducted within a temporary immersion bioreactor system. Our study specifically investigated the impact of *T. longibrachiatum* MD33 on the accumulation of dendrobine in *D. nobile* under controlled experimental conditions.

## Materials and methods

### Test fungal strain and culture conditions

*Trichoderma longibrachiatum* MD33 (hereafter referred to as MD33) was the fungus strain used in this study. It was found on healthy wild *D. nobile* plants collected in Jinshishi, Chishui, Guizhou, China (Sarsaiya et al., 2020a; Sarsaiya et al., 2020b). The strain MD33 has been duly documented and is registered in the NCBI Library under the accession number MN826683. For cultivation purposes, MD33 was nurtured in PD (potato dextrose) broth, maintaining a pH level of 6.5. The cultivation process occurred at a temperature of 28°C over a span of 120 hours, with constant agitation set at 120 revolutions per minute (rpm) (MQD-B2NRG, Shanghai Yuquan Instrument Co. Ltd., Shaghai, China).

### Sample collection and its processing

Sample collection and processing involved the acquisition of *D. nobile* seedlings (8 months old) from the Bioresource Institute for Healthy Utilization (BIHU) at Zunyi Medical University (ZMU) in Zunyi, Guizhou, China. Prior to experimentation, these samples were authenticated to ensure their identity (Zhang et al., 2015; Hu et al., 2016; Yu et al., 2017; Cao et al., 2018). To serve as a growth medium for co-culturing experiments, water agar (0.65% w/v) was used and dispensed into glass bottles. Notably, no supplementary nutrients were introduced into the medium; it consisted solely of double-distilled water and agar. After thorough sterilization of the glass bottles containing the growth medium, the *D. nobile* seedlings were introduced into individual bottles, all under meticulously aseptic conditions.

### Co-culturing endophytic fungi on *D. nobile* seedlings in test bottles

To conduct experiments quantifying dendrobine, we utilized the previously identified endophytic fungal mycelium, *T. longibrachiatum* (MD33) (Sarsaiya et al., 2020a and Sarsaiya et al., 2020b). We processed the contents of endophytic fungal flask cultures to isolate the mycelium suspension. Subsequently, this mycelium suspension was concentrated and introduced into incisions made at the terminal leaves, where they attach to the stem. In parallel, for seedling co-culturing experiments, a sterilized cotton plug was used as a control to establish a baseline reference. To serve as a control, we established triplicate samples under identical conditions but without the addition of endophytic fungal culture. Following the inoculation process, the test co-culturing bottles were relocated to a plant growth light incubator instrument (MGC-300B, Shanghai Yiheng Scientific Instruments Co. Ltd., Shanghai, China), where they were maintained at a temperature of 25°C under a light/dark cycle of 14 hours of light and 10 hours of darkness, with a light intensity of 2000 lux. The evaluation of all test bottles took place after 21 days.

### Co-culturing endophytic fungi with *D. nobile* seedlings in a temporary immersion bioreactor system

To facilitate the co-culturing of endophytic fungi *T. longibrachiatum* (MD33) with *D. nobile* seedlings, approximately 300 seedlings were evenly distributed into each temporary immersion bioreactor. These seedlings were placed in a modified ½ MS culture medium supplemented with ½ MS (2.47 g/L), sugar (30.0 g/L), NAA (0.5 mg/L), and potato extract (30 g/L). The pH of the medium was maintained between 5.8 to 6.0. These bioreactors were then positioned in a well-light growth chamber rack at a constant temperature of 25 ± 2°C with an 11-hour light cycle and a light intensity of 2000 lux. To regulate the immersion of the medium with the seedlings, all bioreactors were connected to the BioF II machine (TIBS, Nanjing BioFunction Co. Ltd., China). The machine controlled the air supply in cycles of 300 seconds, repeated four times with intervals of six hours throughout the day. The experiment was divided into two distinct sets: Set A, where *T. longibrachiatum* (MD33) was introduced, and Set B, which served as the control group without any addition of endophytic fungi. Each set consisted of triplicate bioreactors containing *D. nobile* seedlings. In Set A, approximately 0.25 ± 0.1 grams of wet mycelial growth from pure cultures were introduced into the bioreactors under aseptic conditions. This facilitated the interaction between *T. longibrachiatum* (MD33) mycelium and the *D. nobile* seedlings. In Set B, no endophytic fungal cultures were added to the bioreactors. The interactions between *D. nobile* seedlings and endophytic fungi were observed at seven-day intervals. This included monitoring for mortality rates and changes in the coloration of the *D. nobile* seedlings. After a 21-day cultivation period, the *D. nobile* seedlings were carefully extracted, washed with distilled water, and dissected to separate the stems and leaves for subsequent histopathological analysis. The remaining *D. nobile* seedlings were prepared for dendrobine concentration quantification using the LC-MS technique.

### Extraction of the reference plant dendrobine

To extract dendrobine alkaloids, oven-dried *D. nobile* stem powder was utilized. The dried powder was combined with absolute ethanol and subjected to boiling at 90°C for a duration of 2 hours. Following the extraction process, the mixture was carefully filtered through Whatman filter paper. The filtered sample, obtained through a 0.22 μm filter, was transferred to sample tubes for LC-MS analysis. This analysis involved comparing the peak retention time of the extracted material with a reference standard of dendrobine. The standard dendrobine used was sourced from Chengdu DeSiTe Biological Technology Co., Ltd., located in Chengdu, China, and possessed a purity level exceeding 99%.

### Reference standard dendrobine solution preparation

To create the reference standard dendrobine solution, an initial stock solution was formulated with a concentration of 20.0 μg/ml in chloroform. Dendrobine working standard solutions were prepared at concentrations of 20, 40, 60, 80, and 100 ng/ml from the stock solution. This was achieved through precise dilution of the dendrobine stock solution, using chloroform as the diluent. The dendrobine standard utilized in this procedure was procured from Chengdu DeSiTe Biological Technology Co., Ltd., located in Chengdu, China, and boasted a purity level exceeding 99%.

### Quantification of dendrobine concentration through LC-MS

For the LC-MS analysis, the *D. nobile* seedlings from each bioreactor were subjected to a meticulous sample preparation process. The seedlings were first freeze-dried at a temperature of -40°C for a duration of 10 hours. This freeze-drying process was carried out using a ModulyoD-230 Freeze Dryer from Thermo Electron Corporation, based in Milford, MA. Subsequently, 100 mg of the freeze-dried *D. nobile* seedling parts were finely powdered in a mortar and pestle. The powdered material was then soaked in 50 ml of chloroform for a period of 12 hours while being agitated at 120 rpm. Following this soaking process, 40 ml of the liquid portion was carefully separated from the mixture. The chloroform phase was isolated from the aqueous phase and subjected to evaporation at a controlled temperature of 35°C using a rotary evaporator. This process resulted in the formation of a residual substance. To prepare the sample for analysis, the remaining residue was re-dissolved in 5 ml of chloroform. Prior to the analysis, this solution was meticulously filtered through a 0.22 μ m filter to ensure clarity and purity.

The detection of dendrobine was carried out using an advanced UHPLC system, specifically the Thermo Scientific Dionex UltiMate 3000 from the United States. The system was equipped with a column measuring 150 x 2.1 mm and featuring particles with a size of 1.9 µm. For the mobile phase, a composition of 0.1% formic acid and acetonitrile was employed in a ratio of 95:5 (v/v). The flow rate was set at 0.3 ml/min. Additionally, sheath gas flowed at a rate of 35, auxiliary gas at a rate of 15, and the spray voltage was set at 3.5 kV. The capillary temperature was maintained at 350°C, while the auxiliary gas heater temperature was set at 400°C. During the analysis, a 10 μL volume of the prepared sample was injected. The identification of dendrobine was achieved by comparing retention times, molecular weight (264.195), and LC-MS fragmentation patterns with an authentic chemical reference standard. The dendrobine standard used in this analysis was purchased from Chengdu DeSiTe Biological Technology Co. Ltd. in China, and it possessed a purity level exceeding 99%. To quantify the concentration of dendrobine, a range of different dendrobine concentrations were meticulously prepared. These served as the basis for constructing a standard graph, enabling the accurate measurement of dendrobine concentration in the samples.

### Structural analysis using nuclear magnetic resonance spectroscopy

The TIBS co-cultured *D. nobile* seedling samples were used to study the structure of dendrobine using NMR spectroscopy. The compound under investigation was dissolved in deuterated solvents, including CD_3_OD (δ_H_ 3.31, δ_C_ 49.00), to obtain clear and homogeneous solutions for NMR spectral tests. NMR spectra were acquired on an Agilent DD2400-MR spectrometer, operating at a proton frequency of 400 MHz and a carbon frequency of 101 MHz. The instrument, sourced from Agilent Technologies (Santa Clara, CA), was equipped with state-of-the-art technology to ensure high-resolution spectra. Proton NMR spectra were recorded using a 7 mm NMR tube with the samples dissolved in deuterated solvents. Carbon NMR spectra were acquired using the same samples in 7 mm NMR tubes. The acquired NMR data were processed using Agilent’s proprietary software, and spectra were analyzed using MestReNova (version 14.2.1-27684) to extract relevant information about the chemical structure of dendrobine.

### Statistical analysis

Every experiment conducted in this study was carried out in triplicate, ensuring the reliability and robustness of the results. The data obtained from these experiments were presented as the mean value accompanied by the standard deviation (SD), calculated from the triplicate values. For statistical analysis, Duncan’s multiple range test (DMRT) was employed. This statistical test helps assess significant differences among multiple groups. The entire statistical analysis was performed using the SPSS V16.0 statistical package, a well-established software program developed by SPSS Inc. in Chicago, USA.

## Results

### Quantification of dendrobine in *D. nobile* seedlings using test bottles

**Figure 1:**
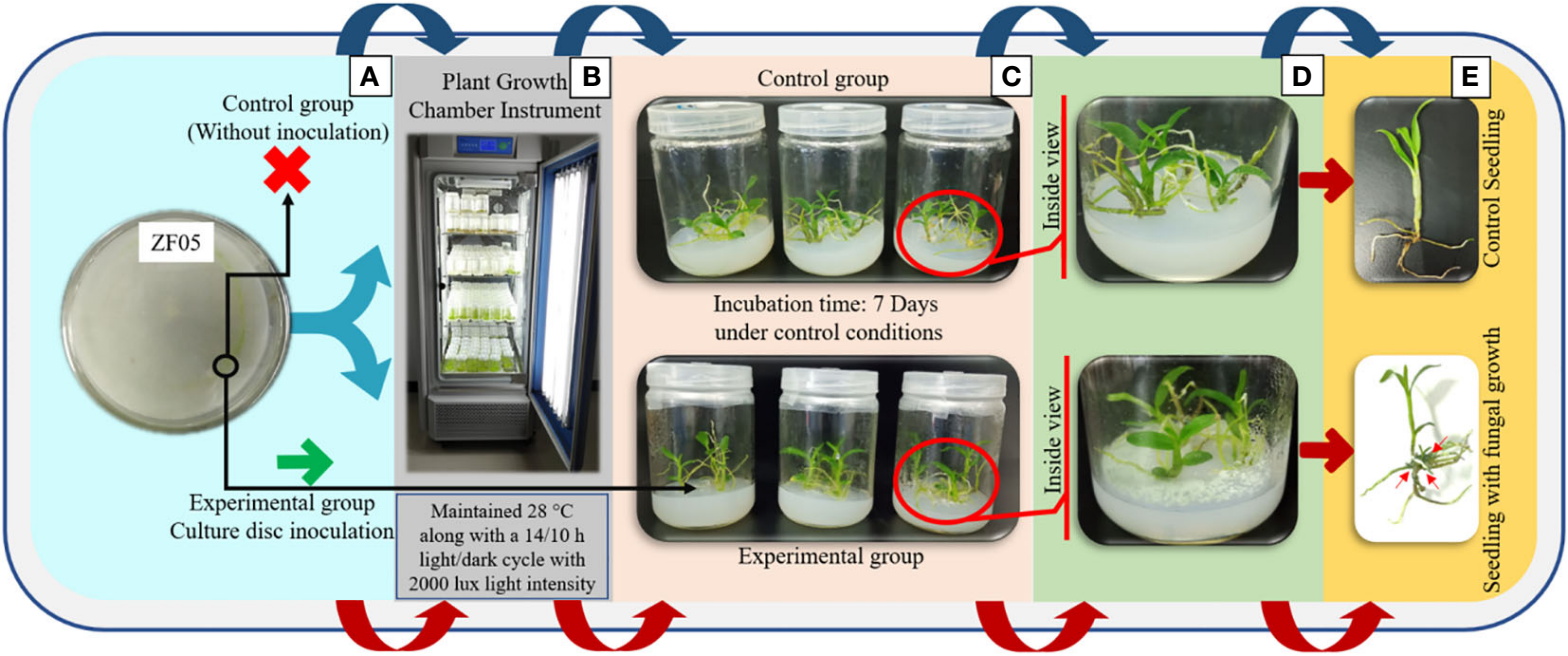
Co-culturing of *Trichoderma longibrachiatum* MD33 with *D nobile* seedlings for high yield of dendrobine concentration. **(A)** Pure culture of *Trichoderma longibrachiatum* MD33; **(B)** Plant growth chamber for maintain the growth conditions; **(C)** Test bottles after 7 days incubation; **(D)** Inside view of test bottles; **(E)** Observation of single seedling.

**Figure 2:**
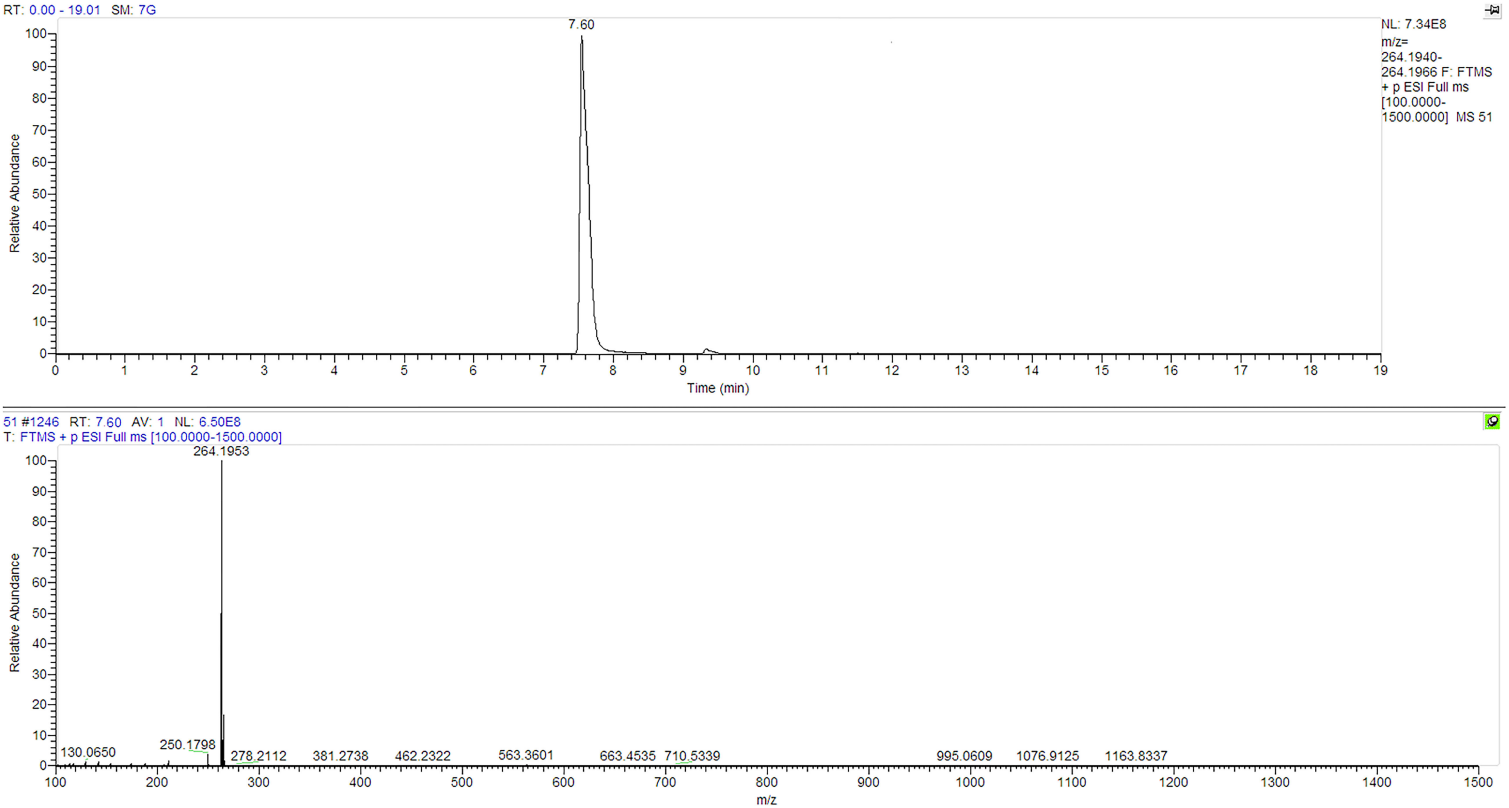
Chemical reference dendrobine (CRD) LC-MS Chromatogram for authentic reference dendrobine peak and molecular weight.

**Figure 3:**
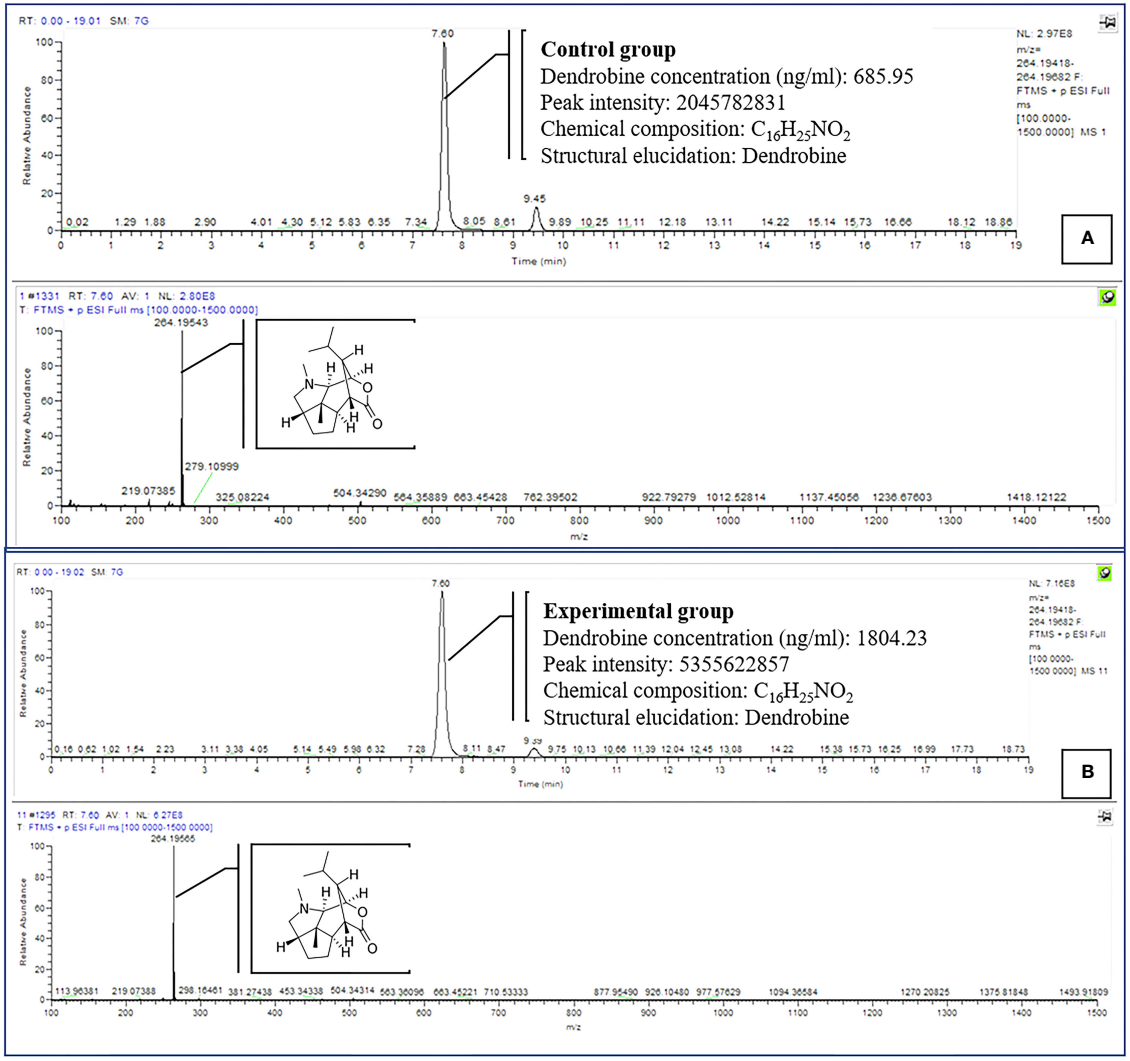
Comparison of dendrobine profiles produced by CGTB **(A)** and EGTB **(B)** *D. nobile* seedlings dendrobine. Tentative LCMS identification of the dendrobine is highlighted in the chromatogram (Molecular weight 264.195).

Dendrobine quantification in *D. nobile* seedlings using test bottles revealed the presence of picrotoxan-type sesquiterpenoids. Dendrobine alkaloids were identified and characterized through unambiguous comparisons with reference standards, incorporating retention time, accurate mass, and MS/MS fragment ions (**Figures 1**, **2**). The chemical composition and details, including retention time and precursor ions, are summarized in **Table 1**, which presents the induced phytodendrobine yield under different conditions in the control (CGTB) and experimental (EGTB) groups. Both groups, utilizing 8-month-old *D. nobile* seedlings, showed similar peak retention times (7.6 ± 0.1 minutes), with dendrobine identified as C_16_H_25_NO_2_ and a molecular weight of 264.195. A bioanalytical approach was used to investigate the effect of cultivating mono-culture of MD33 with and without *D. nobile* seedlings on dendrobine production in solid media test bottles. The UHPLC-MS method, applied to chloroform extracts from *D. nobile* seedlings, illustrated a peak intensity (7.6 ± 0.1 minutes) between the control and co-culture sets (**Figure 3**). Confirmation through LC-MS analysis in positive ion mode, compared with known chemical reference dendrobine, revealed a substantial increase in dendrobine accumulation when MD33 was co-cultured with *D. nobile* seedlings. Co-culture test bottles demonstrated a remarkable 2.6-fold increase in average dendrobine production (1804.23 ng/ml) compared to the control group (685.95 ng/ml) (**Table 1**). The mass of dendrobine in both groups was 264.195, corresponding to the molecular formula C_16_H_25_NO_2_. *D. nobile* seedlings with dendrobine were identified by comparing the peak retention time with that of the chemical reference dendrobine.

**Table 1:** Yield of induced *D. nobile* dendrobine in the test bottles.

Test Group	Plant material	SAM	Detection Technique	Peak retention time (min)	Chemical composition	Structural elucidation	Molecular weight (MW)	Dendrobine concentration (ng/ml)	Fold increased
CGTB	*D. nobile* seedlings	12	UHPLC	7.6 ± 0.1	C_16_H_25_NO_2_	Dendrobine	264.195	685.95	–
EGTB	*D. nobile* seedlings	12	UHPLC	7.6 ± 0.1	C_16_H_25_NO_2_	Dendrobine	264.195	1804.23	2.6*
CRD	–	–	UHPLC	7.6 ± 0.1	C_16_H_25_NO_2_	Dendrobine	264.195	–	–

CGTB, Control Group Test Bottles; EGTB, Experimental Group Test Bottles; CRD, Chemical reference dendrobine; SAM, Seedlings age in months; ng/ml, nanogram per millilitre; *as compared to CGTB.

### Quantification of dendrobine in *D. nobile* seedlings using a temporary immersion bioreactor system

**Figure 4:**
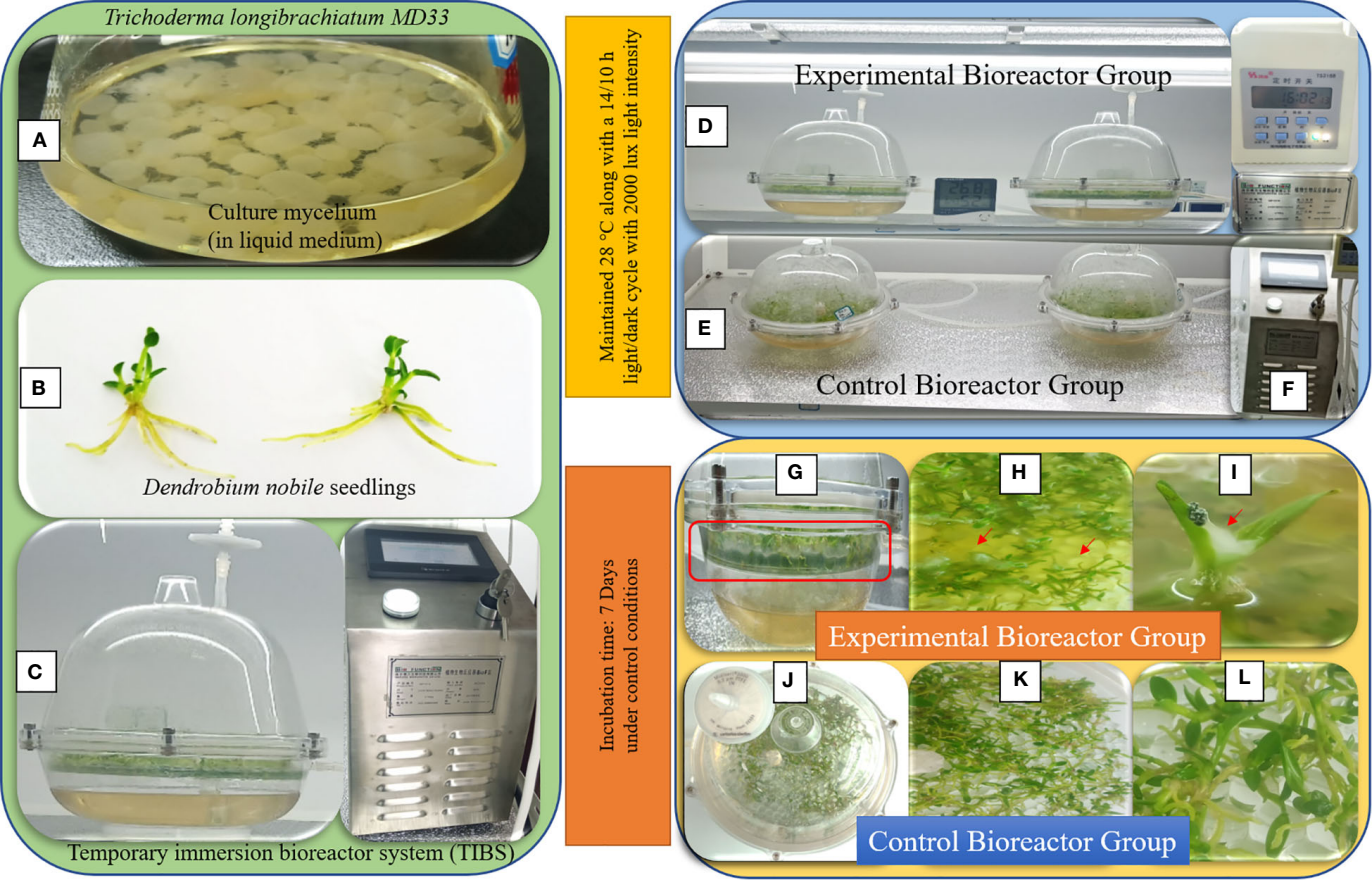
Temporary immersion bioreactor system (TIBS) co-culturing for high yield of *D nobile* seedlings dendrobine. **(A)** *Trichoderma longibrachiatum* MD33 mycelial bolls in the liquid medium; **(B)** Young healthy *D nobile* seedlings; **(C)** Temporary immersion bioreactor system (TIBS) connected to the BioF II machine for supply constant air with constant time; **(D)** Light growth chamber rack for TIBS; G: Side view of EGTIBS bioreactor with clear visible of fungal growth; **(H, I)** *D nobile* seedlings and *Trichoderma longibrachiatum* MD33 mycelium interaction; **(J)** Top view of CGTIBS; **(K, L)** Inside view of CGTIBS.

**Figure 5:**
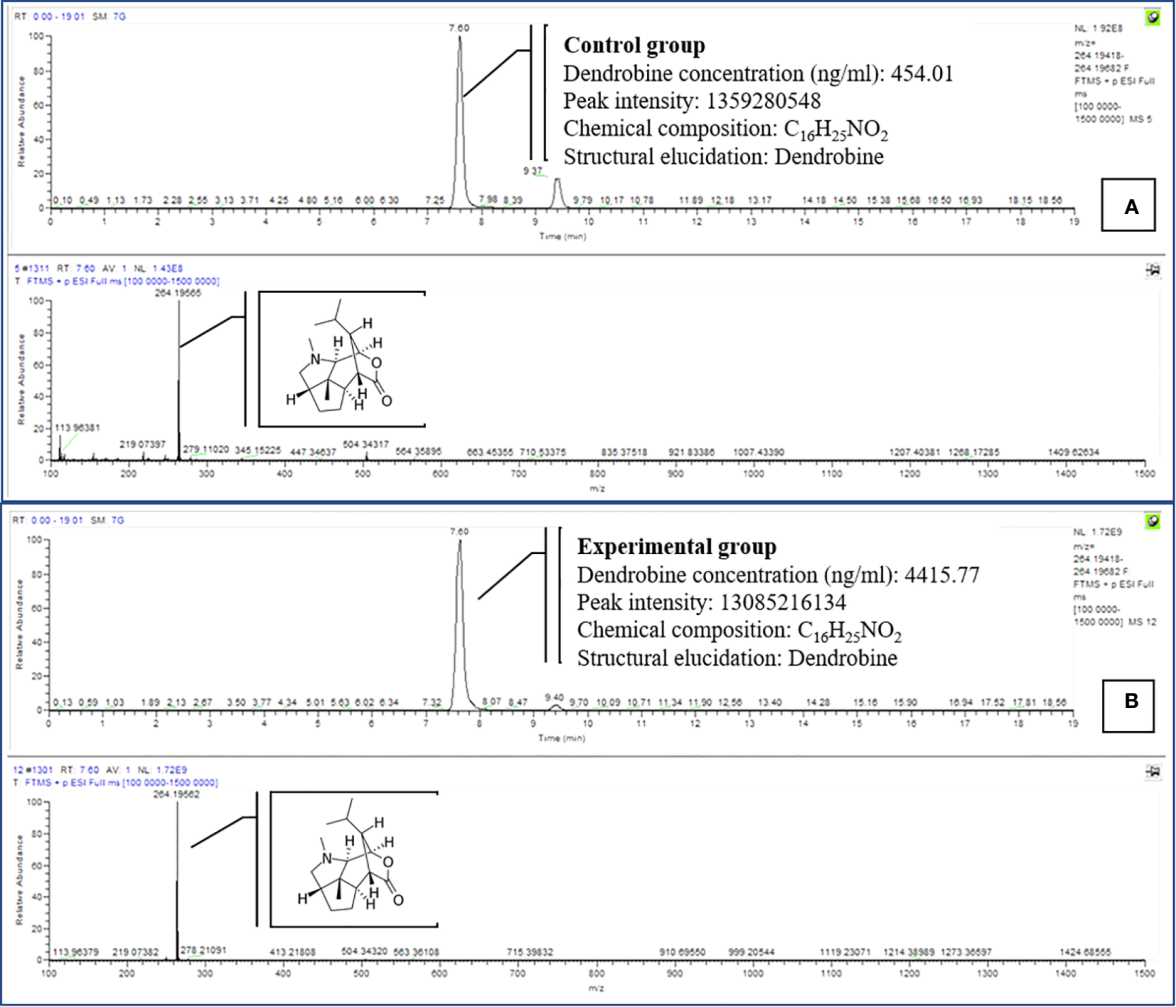
Comparison of dendrobine profiles produced by CGTIBS **(A)** and EGTIBS **(B)** *D. nobile* seedlings dendrobine. Tentative LCMS identification of the dendrobine is highlighted in the chromatogram (Molecular weight 264.195).

We investigated utilizing a temporary immersion bioreactor system (TIBS) to increase dendrobine accumulation in *D. nobile* by co-culturing with MD33. MD33 generally aided *D. nobile* seedling growth. In addition, the experimental and control groups exhibited significantly different dendrobine levels. After 7 incubation days, the experimental group had significantly more dendrobine (**Table 2**; **Figures 4**, **5**) than the control group. In particular, *D. nobile* phytodendrobine accumulation increased 9.7-fold in the experimental group to 4415.77 ng/ml, compared to 454.01 ng/ml in the control group. Chromatography revealed a notable difference in the peak intensity between the MD33 fungal experimental group (13,085,216,134) and the control group (1,359,280,548). The retention times did not differ between the experimental and control groups. Mass spectrometry confirmed the presence of dendrobine in both groups, matching the molecular formula C_16_H_25_NO_2_, with a precise molecular weight of 264.195 in both experimental and control groups. Additionally, the chemical reference dendrobine produced a mass of 264.195 Da with a distinctive peak, and a retention time of 7.6.

**Table 2:** Yield of induced *D. nobile* dendrobine in the temporary immersion bioreactor system (TIBS).

Test Group	Plant material	Liquid medium	SAM	Detection Technique	Peak retention time (min)	Chemical composition	Structural elucidation	Molecular weight (MW)	Dendrobine concentration (ng/ml)	Fold increased
CGTIBS	*D. nobile* seedlings	Modified ½ MS	10	UHPLC	7.6 ± 0.1	C_16_H_25_NO_2_	Dendrobine	264.195	454.01	–
EGTIBS	*D. nobile* seedlings	medium	10	UHPLC	7.6 ± 0.1	C_16_H_25_NO_2_	Dendrobine	264.195	4415.77	9.7
CRD	–	–	–	UHPLC	7.6 ± 0.1	C_16_H_25_NO_2_	Dendrobine	264.195	–	–

CGTIBS, Control Group; EGTIBS, Experimental Group; CRD, Chemical reference dendrobine; SAM, Seedlings age in months; ng/ml, nanogram per millilitre.

### Comparative analysis of co-cultured experiments

Valuable insights into strategies for enhancing dendrobine production were revealed through a comparative analysis of dendrobine concentration in co-cultured *D. nobile* seedlings using an Experiment with Test Bottles (EGTB) and an Experiment with Temporary Immersion Bioreactor System (EGTIBS) (**Table 3**). Moreover, utilizing the prospective endophytic fungus MD33 to co-cultivate *D. nobile* seedlings in the EGTB experiment resulted in a noteworthy 2.6-fold elevation in dendrobine concentration, as confirmed by LC-MS analysis. The effectiveness of MD33 as a facilitator of dendrobine synthesis in *D. nobile* seedlings was demonstrated using this method. However, the outcomes of EGTIBS were even more significant. Dendrobine concentration in seedlings of *D. nobile* cultured in a Temporary Immersion Bioreactor System (TIBS) increased by 9.7-fold compared to that in the control group. The experimental group that utilized TIBS achieved a noteworthy dendrobine concentration of 4415.77 ng/ml, demonstrating the exceptional capacity of this cultivation technique to enhance dendrobine levels. The significant disparity in dendrobine concentrations between the two experimental methodologies highlights the efficacy of the TIBS system for maximizing dendrobine production. Given its exceptional yield potential, future research avenues should consider expanding the TIBS method to commercial dendrobine production because it is particularly advantageous for pharmaceutical and industrial applications. Further investigations into the mechanisms governing the increased production of dendrobine in the TIBS system may yield significant knowledge for optimizing the biosynthesis of dendrobine in *D. nobile*.

**Table 3:** Comparative analysis of EGTB and EGTIBS for *D. nobile* seedlings dendrobine concentration.

Co-cultured group	Host Plant	Test strain	Detection Technique	Dendrobine Yield (ng/ml)	Incubation time (days)	Fold increased *	Fold increased#
EGTB	*D. nobile* seedlings	*Trichoderma longibrachiatum* MD33	UHPLC	1804.23	7	2.6	–
EFTIBS	*D. nobile* seedlings	*Trichoderma longibrachiatum* MD33	UHPLC	4415.77	7	9.7	3.7

*as compared to CGTB and CGTIBS; #as compared to EGTB dendrobine concentration; ng/ml, nanogram per millilitre.

### Structural elucidation of dendrobine

**Figure 6:**
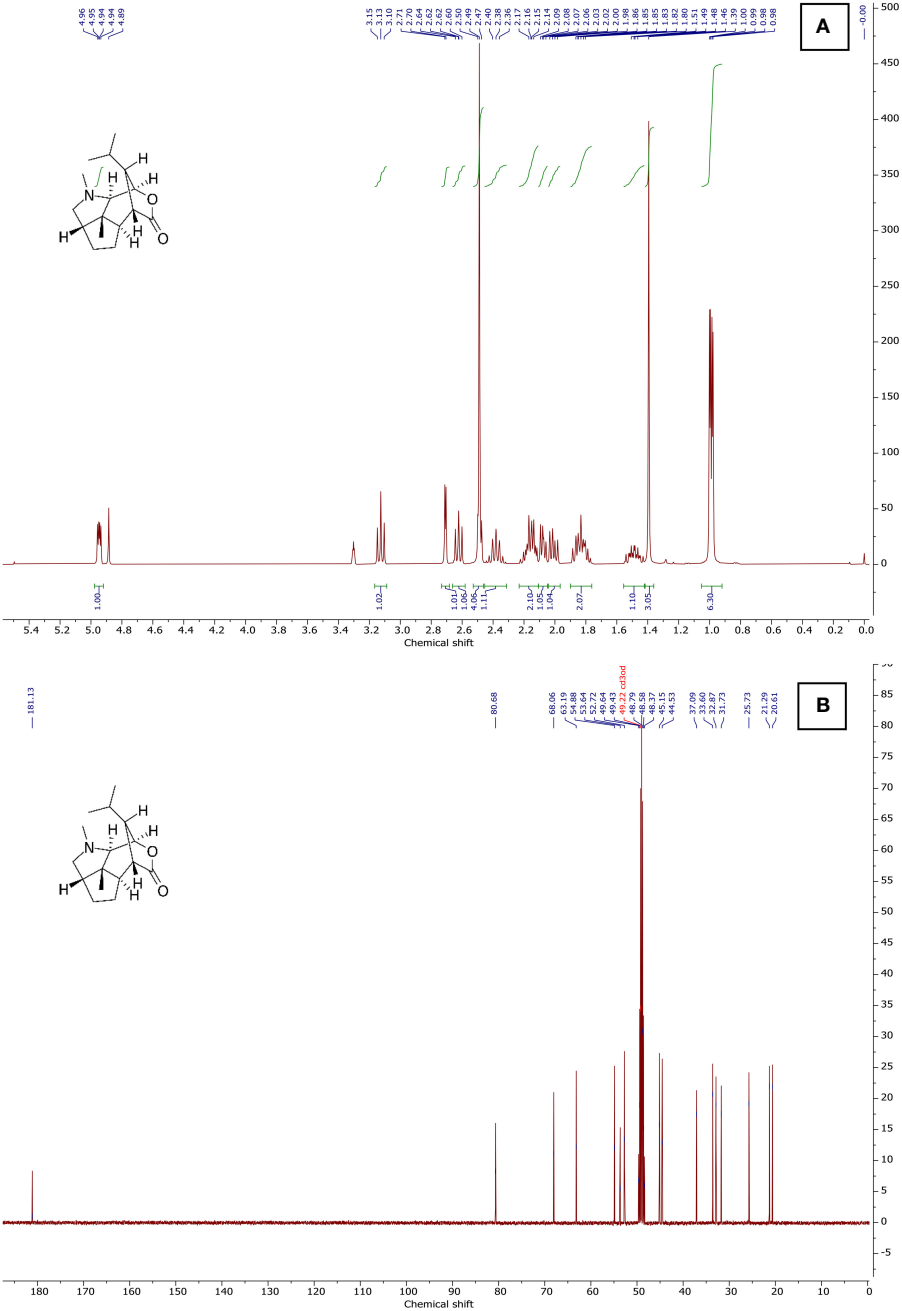
NMR spectra of *Dendrobium nobile* seedling dendrobine using ^1^H and ^13^C NMR. **(A)** ^1^H NMR (400 MHz, CD_3_OD), **(B)** ^13^C NMR (101 MHz, CD_3_OD).

Different proton resonances were observed in the compound’s ^1^H NMR spectrum, which was recorded at 400 MHz using CD_3_OD. The provided ^1^H NMR spectrum reveals distinct chemical shifts and coupling patterns for various proton environments in the organic compound. Peaks A through K exhibit diverse patterns, including singlets (s), doublets (d), triplets (t), and multiplets (m). Notably, peak E appears as a sharp singlet at 2.49 ppm with a high integration value of 2.94, suggesting the presence of three equivalent protons in a unique chemical environment. Peaks G and K, both identified as multiplets, display complex coupling patterns, with peak G showing three protons and an integration value of 3.04, while peak K involves five protons with a substantial integration value of 4.51. The coupling constants (*J*) associated with certain peaks, such as peaks D, I, and K, provide additional information about the coupling interactions between neighboring protons. The provided ^13^C NMR spectrum illustrates distinct carbon chemical shifts and environments within the organic compound. Peaks A to N display sharp singlets (s) with varying chemical shifts, ranging from 80.68 to 20.61 ppm. These peaks correspond to individual carbon atoms in the molecular structure, each characterized by a unique chemical shift and integral value. Notably, peak F stands out with a multiplet (m) at 49.01 ppm, suggesting a carbon environment associated with thirteen protons and an integration value of 13.23. This multiplet indicates a complex coupling pattern, possibly arising from the interaction with nearby protons. Peaks G and O, appearing as singlets at 45.15 ppm and 44.53 ppm, respectively, represent distinct carbon atoms with integral values of 1.27 and 1.32. These findings, combined with the ^1^H NMR results, contribute to a comprehensive understanding of the molecular structure of the compound and serve as a valuable foundation for further analyses in this research context. The ^1^H NMR spectrum in **Figure 6A** shows a chemical shift of the CD_3_OD solvent peak to 3.31, and the ^13^C NMR spectrum shows a chemical shift of the CD_3_OD solvent peak to 49.22 (**Figure 6B**).

## Discussion

Endophytic fungi residing within various tissues and organs of healthy plants maintain a unique association with their host plants without inciting them to stimulate natural compound biosynthesis (Ding et al., 2018; Wang et al., 2023). However, high commercial demand for these fungi has led to their extraction from natural habitats, placing them at risk of extinction. To safeguard and revitalize threatened and rare orchid plants, a reintroduction strategy involving fungal co-culturing becomes imperative (Sarsaiya et al., 2020a). Therefore, it is crucial to identify pure endophytic fungal strains that can stimulate or inhibit the growth of *Dendrobium* seedlings *in vitro*. Research on the endophytic fungi associated with medicinal plants has revealed their potential. Some endophytic fungi can promote the accumulation of bioactive secondary metabolites in their host plants, whereas others can synthesize secondary metabolites similar to those found in their hosts (Sarsaiya et al., 2020b). These characteristics of endophytic fungi present a novel avenue for improving the production of active compounds through co-culturing approaches. Such methods offer new perspectives and techniques for enhancing the accumulation of bioactive components in medicinal plants during co-cultivation while ensuring the sustainable development of traditional Chinese medicine resources (Wen et al., 2022).

The present study explored the mechanism underlying increased dendrobine production, involving intricate interactions between the endophytic fungus and the host plant, especially when cultivated in TIBS. This finding aligns with previous research, suggesting the potential of endophytic fungi to enhance the production of secondary metabolites in medicinal plants (Jia et al., 2016; Ye et al., 2021). The use of the TIBS system proved to be even more promising, resulting in a remarkable 9.7-fold increase in dendrobine concentration after just seven days of incubation. This finding highlights the efficiency of the TIBS system in optimizing dendrobine production. Endophytic fungi form symbiotic relationships with their host plants and influence various physiological and biochemical processes, including the production of bioactive compounds (Verma et al., 2022). *Trichoderma* spp. have been reported to enhance plant growth, induce systemic resistance, and modulate the biosynthesis of secondary metabolites (Dutta et al., 2023). The UHPLC-MS analysis is a robust method for dendrobine identification and quantification. Confirmation of the presence of dendrobine by comparison with reference standards and the chemical reference dendrobine adds credibility to the results. The controlled and optimized environment provided by TIBS seemingly influenced the production and expression of secondary metabolites in MD33 cells compared to the control group. This alteration in secondary metabolite composition could be attributed to specific conditions and nutrient availability within the TIBS system, suggesting that the TIBS approach has a notable effect on the metabolic activity of endophytic fungi and plants (Cao et al., 2024).

*Trichoderma longibrachiatum* (MD33) establishes itself as an endophyte on *D. nobile* by entering the plant through natural openings. This initiated a symbiotic relationship, creating a conducive environment for both organisms. Endophytic fungi contribute to plant well-being by providing nutrients and growth-promoting substances. *D. nobile* serves as a habitat offering nutrients to MD33. The fungus is known for producing secondary metabolites, including dendrobine, which enhances plant biosynthetic pathways, leading to increased dendrobine production (Sarsaiya et al., 2020b). A Temporary Immersion Bioreactor System (TIBS) provides a controlled and optimized environment for co-cultivation with a formulated liquid medium supporting growth. Periodic immersion and perfusion ensure uniform nutrient and oxygen distribution, promoting optimal growth and metabolic activity (Zhang et al., 2018; Hwang et al., 2022). Employing UHPLC-MS analysis, the study quantifies dendrobine, revealing a substantial increase in concentration in the TIBS approach compared to traditional test bottles. The researchers found that the TIBS is effective in quickly multiplying a significant number of *Dendrobium* seedlings. This makes it well-suited for large-scale production of dendrobine-type total alkaloids (DTTAs) and supports robust plant growth (Zhang et al., 2022; Cao et al., 2024). This highlights the scalability and commercial potential of TIBS for dendrobine production.

The utilization of LC-MS analyses has revealed notable variations in metabolite expression during dynamic interactions between the phytopathogen *Fusarium verticillioides* and the endophyte *Ustilago maydis*. Similarly, co-culturing *Paraconiothyrium variable* with *F. oxysporum* led to the induction of 12 metabolites, as reported by Serrano et al. (2017). These findings emphasize the significance of investigating the potential of Orchidaceae fungal endophytes (OFEs) as valuable sources of bioactive products, with implications for both agriculture and pharmaceuticals (Gupta et al., 2023). However, it is essential to acknowledge that previous research on OFEs-based products is relatively limited, necessitating further investigations into various facets, including screening procedures, extraction methods, separation techniques, and structural elucidation. The continuous symbiotic growth between plants and fungal endophytes makes them promising candidates for mediating bioproduct opportunities through enzymatic processes that alter their biochemical composition. Fungal endophytes can exert either stimulatory or inhibitory effects on host Orchidaceae metabolism. Additionally, the diversity within the host varieties may also play a pivotal role in influencing the profile of secondary metabolic bioproducts in OFEs, as underscored by studies conducted by Sarsaiya et al. (2019) and Adeleke and Babalola (2021).

Fungal-plant interactions involve complex signaling pathways. *Trichoderma* spp. produce signaling molecules and enzymes that modulate plant gene expression. The activation of specific genes involved in alkaloid biosynthesis could be a result of these signaling interactions, leading to increased dendrobine production (Druzhinina et al., 2011). Such symbiosis may result in a metabolic priming effect, in which the plant reallocates resources for secondary metabolite production, including alkaloids. The presence of endophytes such as MD33 may contribute to an altered metabolic profile in *Dendrobium*, favoring metabolite synthesis (Chen et al., 2022). The mechanism by which fungi enhance alkaloid production in plants is likely multifaceted and involves a combination of growth promotion, defense induction, signaling modulation, nutrient exchange, biosynthetic pathway activation, and epigenetic modifications. Further research is needed to unravel the specific molecular interactions between *D. nobile* and *T. longibrachiatum* (MD33) and to identify the key factors influencing the observed increase in dendrobine production. While acknowledging the known capabilities of *Trichoderma* species in enhancing plant growth and modulating biosynthetic pathways, the findings suggest that the effects of MD33 on dendrobine metabolic pathways should be explored. This included elucidating the specific molecular interactions between *D. nobile* and MD33 and identifying key factors influencing the observed increase in dendrobine production. Such insights are crucial for optimizing dendrobine biosynthesis and may have broader implications for cultivating medicinal plants with enhanced bioactive compound yields, emphasizing the multifaceted nature of symbiosis between endophytic fungi and host plants in influencing secondary metabolite production.

The intricate orchestration of dendrobine production involves the integration of various biogenetic routes, with the mevalonate (MVA) and MEP pathways playing pivotal roles. Qian et al. (2021) explored fungal molecular adaptation and revealed a complex genetic landscape with 1,024 differentially expressed genes, suggesting a metabolic symphony controlled by backbone post-modification enzymes and transcription factors. Furthermore, this study highlights the potential regulatory role of miRNAs in modulating dendrobine biosynthesis by targeting key enzymes such as AACT, MK, DXR, and HDS. This co-cultivation strategy encourages a broader perspective, extending beyond genetic expression, to elucidate the intricate relationships between mutagenesis, genetic networks, and metabolite dynamics. Additionally, the findings of Mou et al. (2021) challenged the conventional primacy of the MVA and MEP pathways, emphasizing the significance of AACT, PMK, and MVD genes in the MVA pathway and the upregulation of DXR and DXS genes in the MEP pathway after methyl jasmonate treatment. A comprehensive approach integrating genetic, miRNA, and pathway studies advances our understanding of dendrobine biosynthesis and opens avenues for biotechnological applications and targeted metabolic engineering to enhance dendrobine yield. Gong et al. (2022) identified enzyme genes that contribute to the groundwork for a comprehensive investigation of the dynamic interactions among key enzymes, shedding light on the complex regulatory mechanisms governing dendrobine biosynthesis pathways in a co-cultured system. This study significantly enhances our knowledge of dendrobine biosynthesis within the TIA family, offering potential insights for optimizing production and applications in the field of plant biology.

The structural elucidation of dendrobine through the combined analysis of ^1^H and ^13^C NMR spectra provided valuable insights into the molecular composition and bonding patterns of this natural product. The diverse array of proton environments revealed in the ^1^H NMR spectrum reflects the complexity of the dendrobine chemical structure (Liu et al., 2020). Dendrobine exhibits structural similarity to the potent convulsant picrotoxinin, as elucidated by Connolly and Heathcock (1985) using proton and ^13^C nuclear magnetic resonance (NMR) spectroscopy. This analysis revealed that dendrobine formed a single stereoisomer at the newly created stereocenter. The ^13^C NMR data showed 16 carbon signals, including four methyls (one N-CH_3_), three methylenes, four methines, two quaternary carbons, two carbonyl carbons, and an oxygenated olefinic tertiary carbon. The ^13^C NMR spectrum recorded at 101 MHz in CD_3_OD supported these results. It showed clear carbon resonances with chemical shifts at 80.68 to 20.61 ppm, creating a singlet. Yang et al. (2018) provided additional insights into the NMR structure of dendrobine compared to the known dendrobine-type alkaloid dendrobine. The correlations from H3-16 (δ_H_ 2.96) to C-11 (δ_C_ 52.1, t) and C-15 (δ_C_ 171.6, s), and from H2-11 (δ_H_ 3.42, 2.93) to C-15, indicated a linkage between C-11 and C-15 via a nitrogen atom, resulting in the formation of a seven-membered lactam moiety. Analyzing the ^1^H-NMR spectrum, four dendrobine methyl signals were identified. Peaks A to K exhibit diverse patterns, including singlets, doublets, triplets, and multiplets, each indicative of unique chemical environments. The ^13^C-NMR spectrum displayed all 16 carbons, with C-12 readily identified in the carbonyl region. The quaternary carbon signal in the spectrum was assigned to C-11. Additionally, an oxygen-bearing CH signal was attributed to C-9, and two deshielded CH signals were assigned to C-7 and C-10, respectively (Wang et al., 1985). These combined NMR results provide a solid foundation for ongoing research, offering valuable insights into the intricate molecular architecture of the compound and paving the way for further in-depth analyses and applications.

## Conclusion

In this study, we embarked on a journey to harness the potential of endophytic fungi, specifically *T. longibrachiatum* (MD33), for the enhanced production of dendrobine, a valuable alkaloid found in *D. nobile*. Our findings illuminate the transformative impact of mono-culturing these endophytic fungi within the confines of a Temporary Immersion Bioreactor System (TIBS) and traditional test bottles. In the test bottle experiment (EGTB), the co-cultivation of *D. nobile* seedlings with *T. longibrachiatum* (MD33) led to a commendable 2.6-fold increase in dendrobine concentration. This result underscores the potential of *T. longibrachiatum* (MD33) as a facilitator for dendrobine production within a controlled environment. However, the real breakthrough came with the application of the Temporary Immersion Bioreactor System (TIBS) in the EGTIBS experiment. Here, co-culturing *D. nobile* seedlings with *T. longibrachiatum* (MD33) resulted in a remarkable 9.7-fold increase in dendrobine concentration. The dendrobine content in the TIBS experimental group soared to an impressive 4415.77 ng/ml, far surpassing the control group’s yield. This striking difference underscores the immense potential of the TIBS system for optimizing dendrobine production. Our study exemplifies the power of endophytic fungi to enhance the accumulation of valuable metabolites in medicinal plants like *D. nobile*. This newfound knowledge opens doors to potential applications in pharmaceuticals and other industries. Additionally, our research suggests that scaling up the TIBS approach for commercial dendrobine production holds great promise. As we conclude this endeavor, we look forward to future research directions with optimism. Further investigations could delve into refining the TIBS system, optimizing co-culturing conditions, and exploring the pharmacological potential of the increased dendrobine yield. Additionally, understanding the underlying mechanisms responsible for enhanced dendrobine production within the TIBS system could unlock even greater possibilities for the cultivation of *D. nobile* and other medicinal plants. The comprehensive analysis of the ^1^H and ^13^C NMR spectra of dendrobine has provided essential structural information, allowing for the identification of various proton and carbon environments within the molecule. The elucidated structural features contribute to a deeper understanding of dendrobine molecular architecture, laying the foundation for further investigations into its biological activities and potential synthetic applications. Our study marks a significant step forward in the sustainable utilization of natural resources and the advancement of biotechnological applications in the realm of medicinal plant metabolites.

## Data availability statement

The original contributions presented in the study are included in the article/**Supplementary Material**. Further inquiries can be directed to the corresponding authors.

## Author contributions

SS: Conceptualization, Data curation, Formal analysis, Funding acquisition, Investigation, Methodology, Resources, Validation, Writing – original draft, Writing – review & editing. AJ: Data curation, Formal analysis, Investigation, Resources, Visualization, Writing – review & editing. FS: Formal analysis, Writing – review & editing. MY: Resources, Writing – review & editing. MP: Resources, Writing – review & editing. QJ: Formal analysis, Resources, Visualization, Writing – review & editing. QG: Project administration, Supervision, Writing – review & editing. QW: Project administration, Supervision, Writing – review & editing. XQ: Resources, Visualization, Writing – review & editing. JS: Conceptualization, Funding acquisition, Investigation, Project administration, Resources, Supervision, Writing – review & editing. JC: Investigation, Project administration, Resources, Supervision, Writing – review & editing.
