# Supplementary figures and images for: Enhancing dendrobine production in Dendrobium nobile through mono-culturing of endophytic fungi, Trichoderma longibrachiatum (MD33) in a temporary immersion bioreactor system
Source: Front Plant Sci. 2024 Jan 29;15:1302817. doi: 10.3389/fpls.2024.1302817 (PMC10859523; doi:10.3389/fpls.2024.1302817)

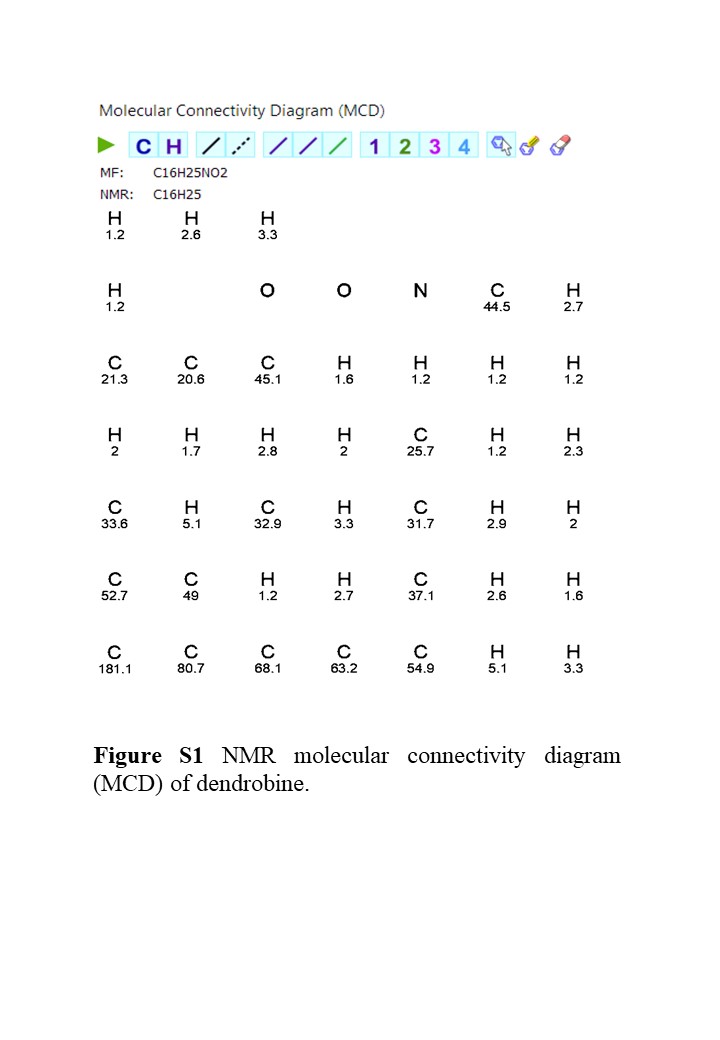

Supplement: Supplementary file 1 [file Image_1.jpeg]
